# Psychosocial impact of surgical complications and the coping mechanisms among surgeons in Uganda and Eastern Democratic Republic of the Congo

**DOI:** 10.1371/journal.pgph.0003180

**Published:** 2024-04-29

**Authors:** Franck Katembo Sikakulya, Joshua Muhumuza, Bives Mutume Nzanzu Vivalya, Simon Binezero Mambo, Larrey Kasereka Kamabu, John Kasereka Muteke, Justin Paluku Lussy, Michel Kalongo Ilumbulumbu, Tapem Emmanuel, Sonye Magugu Kiyaka, Alpha Kavuyiro, Claude Mukandirwa, Hervé Monka Lekuya, Bienfait Mumbere Vahwere, Xaviour Francis Okedi, Claude Kasereka Masumbuko

**Affiliations:** 1 Faculty of Clinical Medicine and Dentistry, Department of Surgery, Kampala International University Western Campus, Ishaka-Bushenyi, Uganda; 2 Faculty of Medicine, Université Catholique du Graben, Butembo, Democratic Republic of the Congo; 3 Faculty of Clinical Medicine and Dentistry, Department of Psychiatry and Mental Health, Kampala International University Western Campus, Ishaka-Bushenyi, Uganda; 4 Youth Alliance for Reproductive Health, Goma, Democratic Republic of the Congo; 5 Allied Health Sciences Department, Kampala International University Western Campus, Ishaka-Bushenyi, Uganda; 6 Department of Surgery, Division of Neurosurgery, Makerere University CHS, Kampala, Uganda; 7 Faculty of Medicine, Université de Goma, Goma, Democratic Republic of the Congo; 8 Department of Surgery, Makerere University, Kampala, Uganda; 9 Faculty of Medicine, Ghent University, Ghent, Belgium; University of Global Health Equity, RWANDA

## Abstract

We aimed to assess the psychosocial impact from postoperative complications on the surgical workforce and the coping mechanisms they use following these complications in Uganda and Eastern Democratic Republic of the Congo (DRC*)*. This was a cross-sectional multi-center study conducted from first February 2022 to 31^st^ March 2022 in the preselected main teaching hospitals of Uganda and Eastern DRC. We surveyed the surgical workforce (practicing surgeons, Obstetrician-Gynecologists, and residents in surgery/ Obstetrics-Gynecology) who had experienced postoperative complications in their career. Data was analysed using SPSS version 23. One hundred ninety-eight participants responded to the questionnaire. Worry about patient and reputation were the commonest psychological impacts in 54.0% and 45.5% of the participants respectively. Majority of the participants (55.1%) used positive coping mechanisms with a positive impact on their practice (94.4%). Being a female doctor (AOR = 2.637, CI 1.065–6.533, P = 0.036), worrying about reputation (AOR = 3.057, CI = 1.573–5.939, P = 0.001) and guilt after a complication (AOR = 4.417, CI = 2.253–8.659, P = <0.001) were predictors of a negative coping mechanism. Postoperative surgical complications continue to cause a huge psychological impact on the operating doctors in Uganda and the Eastern DRC. Female doctors, those that worry about the reputation and those that feel guilty following a complication should be given more support and guidance by peers when surgical complications occur to their patients.

## 1. Introduction

Surgical complications are a public health concern worldwide and occur in 8 to 12% of surgeries done [1]. Both the patients and the surgeons are concerned by surgical complications which not only affect the patient-doctor relationship [2] but also may affect the clinical performance of the surgeon [3]. In most cases, proper preoperative preparation of patients including their psychological and physical well-being impact patients’ response to surgical complications. Surgical complications due to medical errors cause important stress to the operating surgeon, who has the responsibility of providing healthcare and explanation to the patients and their relatives regarding the surgical complications despite the surgeon’s own psychological status. Evidence shows that the impaired psychological well-being of the operating surgeon is a major cause of poor outcomes of the surgery [4]. Hitherto, few efforts have targeted the provision of professional and personal support to the operating surgeon (“the second victim of a surgical procedure”), the surgeon’s daily social living is affected by the operation more especially when complications occur [1,2,4].

Evidence has shown that the operating surgeon usually experiences impaired quality of life, depression, and anxiety which disorders result in recurrent surgical mistakes [3]. A previous study found that poor surgical outcomes were the greatest predictor of burnout among 7905 American surgeons [5]. The surgeons that had burn out presented with adverse emotional influence following surgical complications such as anxiety, guilt, interference with professional and leisure activities, coping mechanisms characterized by a limited discussion with colleagues, alcohol and substance abuse, a perception that emotional distress would be perceived as a constitutional weakness, and changes in clinical practice as well as participating in root-cause analysis [5]. Moreover, the psychosocial impact on a surgeon after a surgical operation with complication is linked to years of clinical practice, the worked hours per week including the night calls, and the age of the operating surgeon [4]. In fact, the factors differentiating the impact are the severity of surgical complication, the seniority of the surgeon; and the reported emotions that persist across the surgeon’s journey towards recovery. Proper management of the stress related to the surgical complications was correlated largely to constructive behaviors and appropriate clinical practice [1,6].

Surgical complications are partly attributed to the paucity of the trained surgical workforce and the poor post-operative management in developing countries [7], with the risk of death doubled in developing countries. In these countries, health worker shortages hinder the ability to cope with the demand of the surgical disease burden’ which leads to increased workload for most surgeons (probably about 80% in most developing countries) [8] as shown in Uganda and Democratic Republic of the Congo (DRC) [9]. In this context, poor remuneration, the overwhelming patient to surgeon ratio and increased rate of surgical complications impact on the surgeon’s outcomes over time [10].

Early identification of patients at high risk for postoperative complications may help decrease mortality and help surgeon burnout [11]; Providing psychosocial support to surgeons may decrease burnout. However, none of these have been studied among surgeons practicing in Uganda and DRC to understand how they respond to surgical complications which are on the rise in these countries [12,13]. This study was done to assess the psychosocial impact of surgical complications on the operating surgeon and the coping mechanisms they use after the complicated surgeries.

## 2. Methods

### Study design and setting

**Fig 1:**
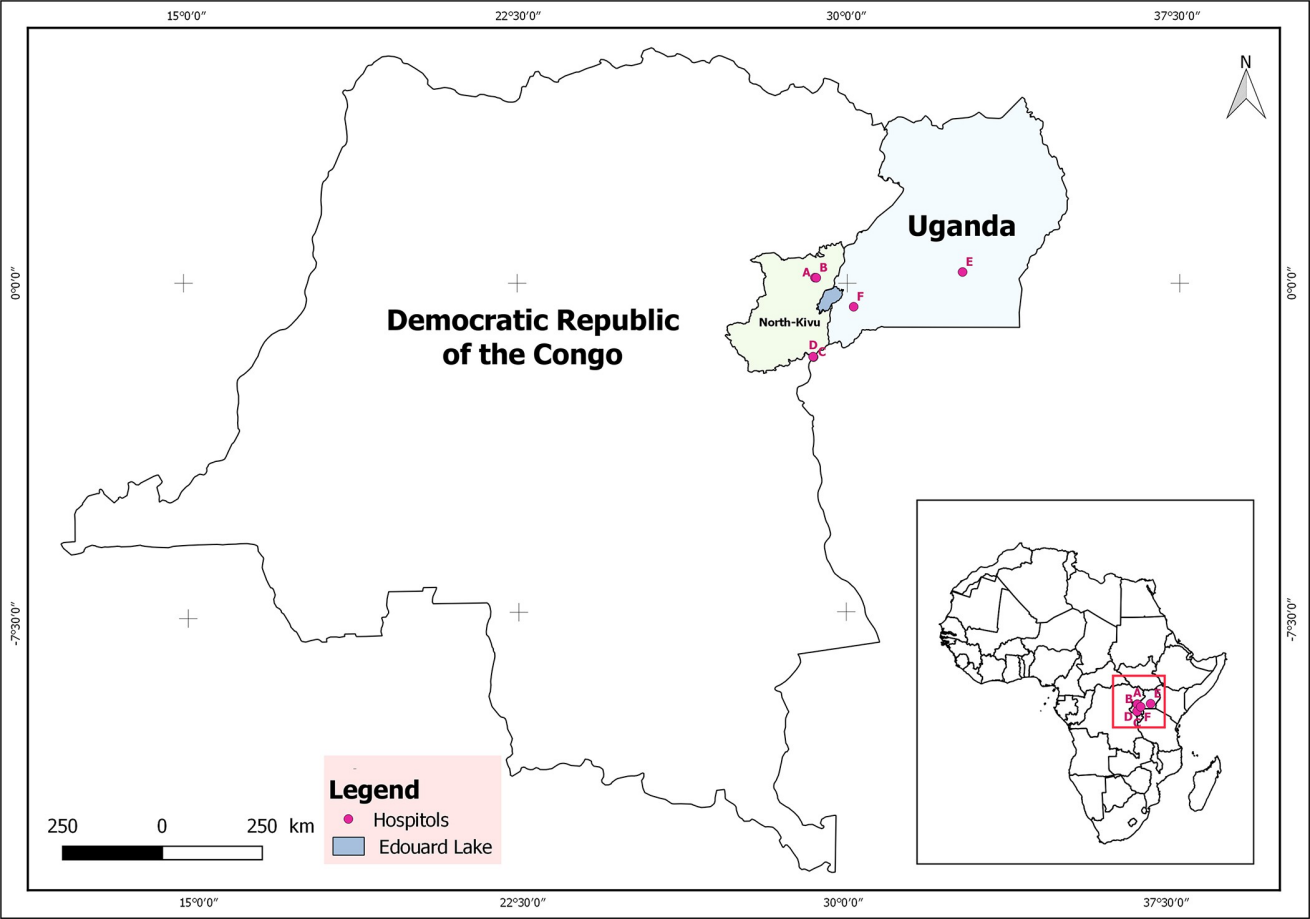
Map of the region with location of the different hospitals in DRC and Uganda performed using QGIS software with RGC (Referentiel Geographique Commun) for shapefile of DRC and North-Kivu (https://www.rgc.cd/).

This was a cross-sectional multi-center study carried out in the main teaching hospitals of Eastern DRC and Uganda. As per the Fig 1 attached, in DRC, Cliniques Universitaires du Graben of the Université Catholique du Graben (A), Matanda Hospital (B), Heal Africa (C) and Hopital Provincial in North Kivu DRC (D). In Uganda, Mulago National referral Hospital (E) and Kampala International Hospital (F) were considered as study areas. These hospitals were chosen because they are the key points of treatment in the countries and receive referrals from all the health facilities within the regions. The hospitals were also well-equipped in terms of human resources and facilities to provide high-quality surgical services for many patients in need.

### Study participants

Using convenience sampling technique, we collected data from the surgical workforce (practicing surgeons, Obstetrician-Gynecologists, and residents in surgery/ Obstetrics-Gynecology) who had experienced postoperative complications from their patients and who consented to participate in the study from the preselected teaching hospitals. Considering 400 as effective surgical workforce based on records of the preselected teaching hospitals, using the Krejcie & Morgan (1970) table for estimating sample size for a given population for easy reference, a minimum sample size of 196 HCWs was considered at a 95% level of confidence and a margin of error of 0.05. Based on the idea that a questionnaire’s non-completion rate could be as high as 10%, this was increased to 216.

### Data collection, instruments and variables

Data was collected for a period of two months from first February 2022 to 31^st^ March 2022. Before beginning to respond to the structured questionnaire, participants gave consent. A structured questionnaire written in English and in French was composed of 13 items based on a published tool validated by *Biggs, S.,et al. [14]*. The 13 questions were divided into four main categories: involvement in complications, the impact of complications on one’s personal and professional life, accessible, used, and wanted support, and obstacles to getting support. The coping mechanisms were classified as positive (internalizing, discussing with colleagues, patient or relatives, patient management and getting on with life) or Negative (Blaming self or others, alcohol use, self-destruction and disassociation). The impact to surgical practice was also classified as positive (reflective practice, being extra cautious and risk averse) or Negative (withdraw from surgical practice). There were both closed and open multiple-choice and yes/no items. Not all responses were necessary for the survey to be considered complete.

### Data processing and analysis plan

Data was acquired, cleaned, and exported into SPSS version 23 for analysis after being entered into Microsoft Excel. The baseline characteristics, the complications encountered, the physiological impact and the coping mechanisms were established using descriptive statistics. Categorical variables were summarized using frequencies and percentages and presented in either a tabular form, bar graph or pie chart. To determine the predictors of negative coping mechanisms, bivariate and multivariate analysis was done using binary logistic regression. All variables with scientific plausibility and those with a p value less than 0.2 at bivariate analysis were analysed at multivariate level using backward logistic regression. Variables in the final model with a p value less than 0.05 were considered to be significant predictors of negative coping mechanisms.

### Ethical considerations

Ethical clearance for the study was obtained from the medical ethics committee of Heal Africa Tertiary Hospital in DRC (001/HA/CEM-HATS/G-NK/2022). Before HCWs participated in the study, permission to visit health facilities was sought from the management of various health facilities as well as the pertinent local health authorities. Participants were anonymous, participation was voluntary, and there were no rewards for participating.

## 3. Results

### Sociodemographic characteristics of participants

Majority of the participants were from Uganda (72.7%), Male (83.3%), and residents (58.1%) from the public sector (56.6%) (Table 1).

**Table 1:** Baseline characteristics of study participants.

Characteristic	Statistic	
**Age**	Mean = 35.929, SD = 8.4538, Min = 25.0, Max = 75.0	
**Years of Experience**	Mean = 7.75, SD = 6.78, Min = 1.0, Max = 45.0	
	**Frequency**	**Percentage**
**Country**		
Uganda	144	72.7
DRC	54	27.3
**Sex**		
Male	165	83.3
Female	33	16.7
**Religion**		
Christian	172	86.9
Non-Christian	26	13.1
**Grade**		
Specialist	45	22.7
Resident	115	58.1
General Practitioner	38	19.2
**Position**		
Hospital Director	4	2.0
HOD	15	7.6
Practitioner	179	90.4
**Sector**		
Public	112	56.6
Private	86	43.4
**Only Surgeon in department**		
No	171	86.4
Yes	27	13.6
**Full time**		
Full Time	172	
Part Time	26	
**Specialty**		
General surgery	94	47.5
Obstetrics and Gyn	58	29.3
Orthopedic Surgery	32	16.2
Neuro Surgery	2	1.0
Other	12	6.1

### Complications related to surgery as reported by participants

**Fig 2:**
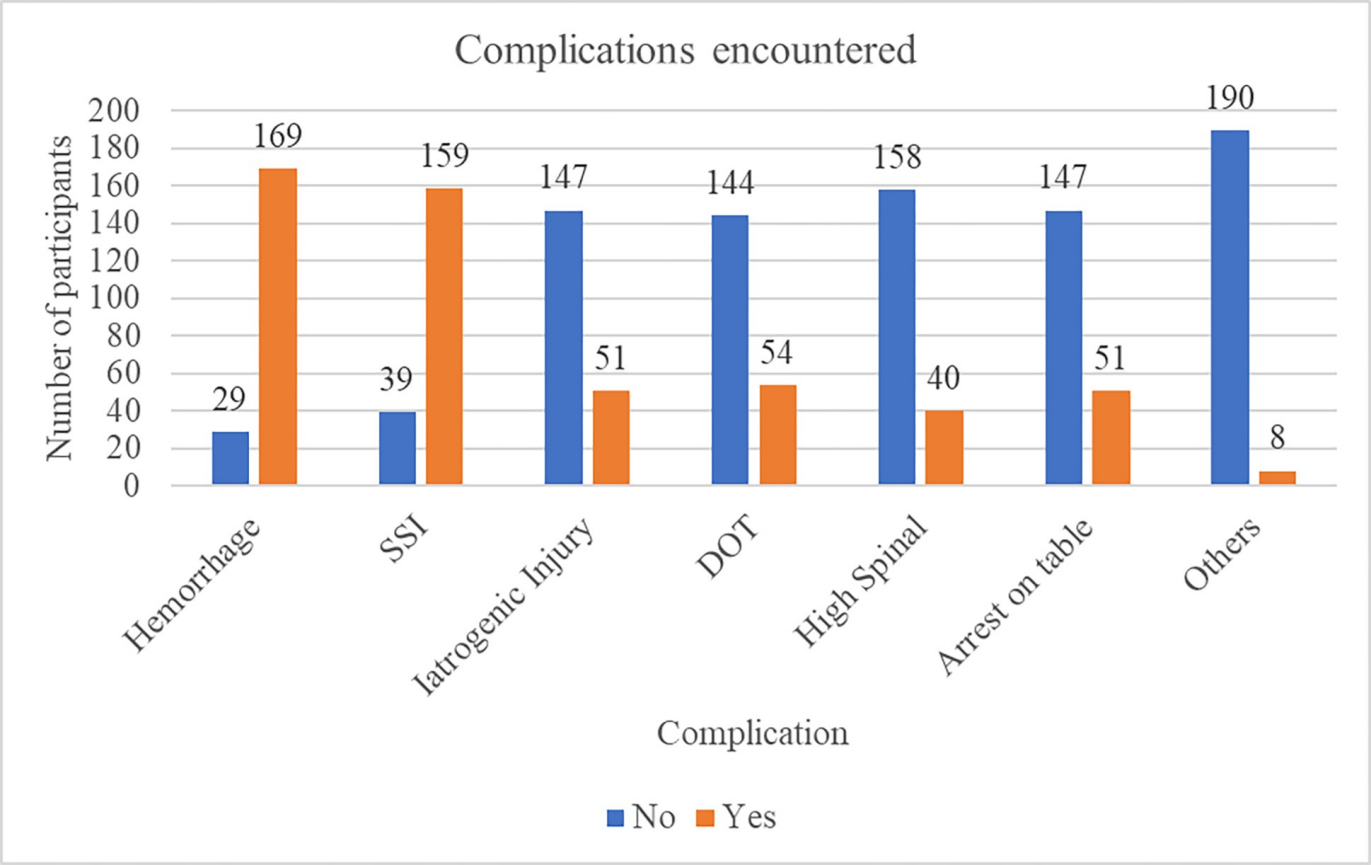
The complications encountered. SSI: Surgical site infection; DOT: Death on table.

Majority of the participants had encountered less than 50 complications (93.4%). The most common complications seen were hemorrhage (85.4%) and SSI (80.3%). Death on table was experienced by 27.3% of the participants (Fig 2).

### Emotional impact seen following a complication

**Fig 3:**
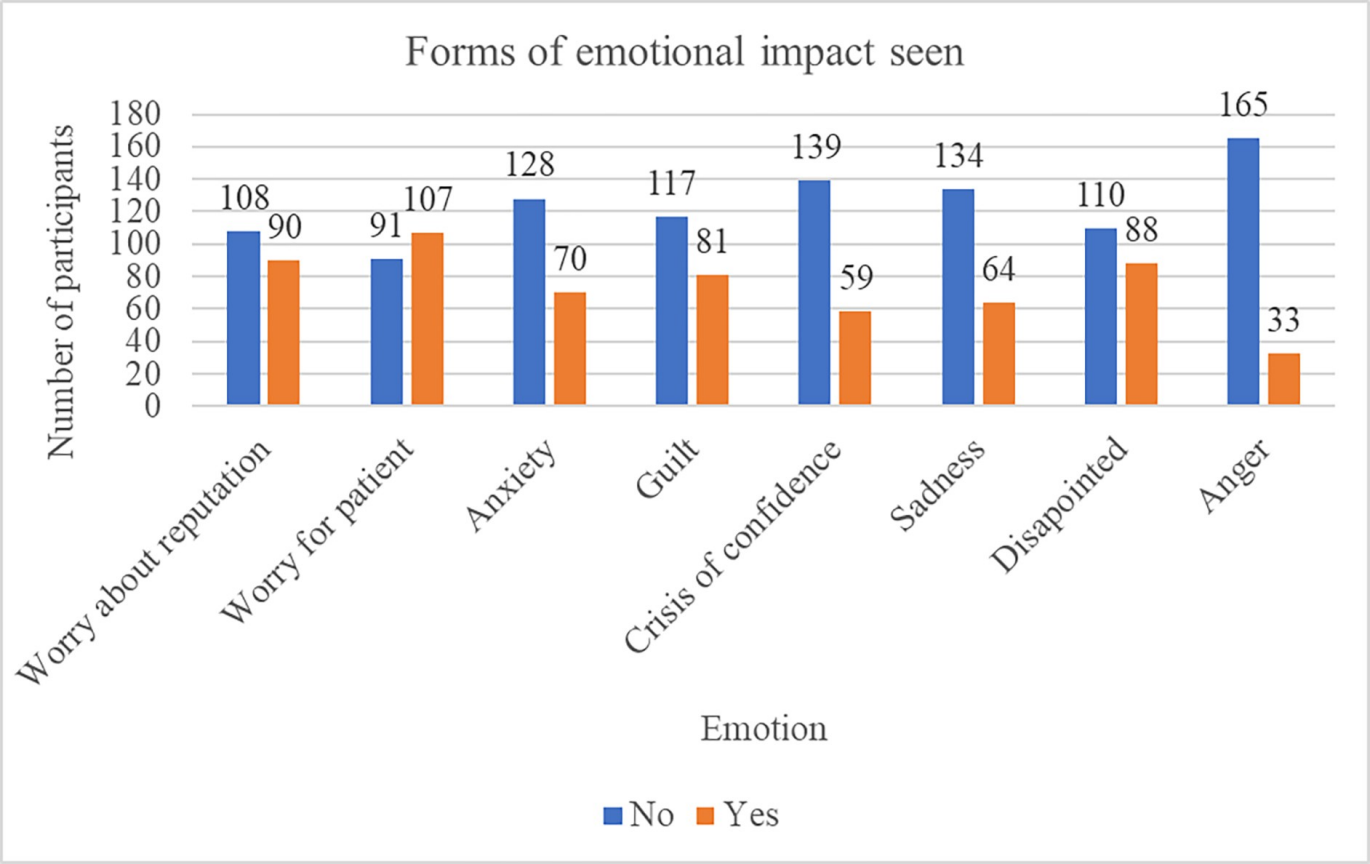
Forms of emotional impact seen following a complication.

Worry about patient and reputation were the commonest; seen in 54.0% and 45.5% of the participants respectively. Participants reported to feel guilty in 40.9% and in 44.4% disappointed following a complication (Fig 3).

### Mechanisms used by the participants following a surgical complication

**Fig 4:**
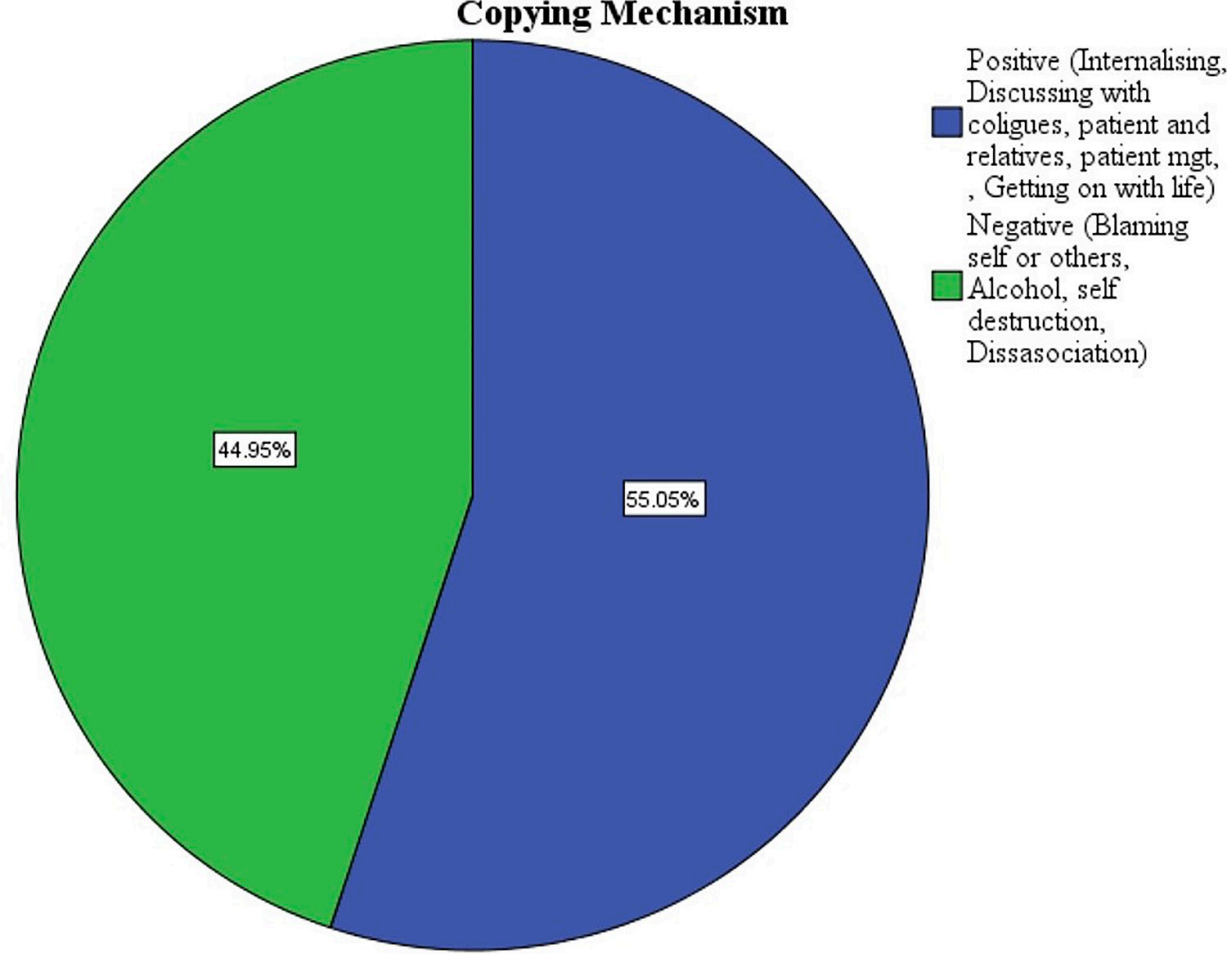
Coping mechanisms used by the participants following a complication.

Majority of the participants (55.1%) used coping mechanisms that were considered positive (Fig 4).

### Impact on surgical practice of participants following surgical complications

**Fig 5:**
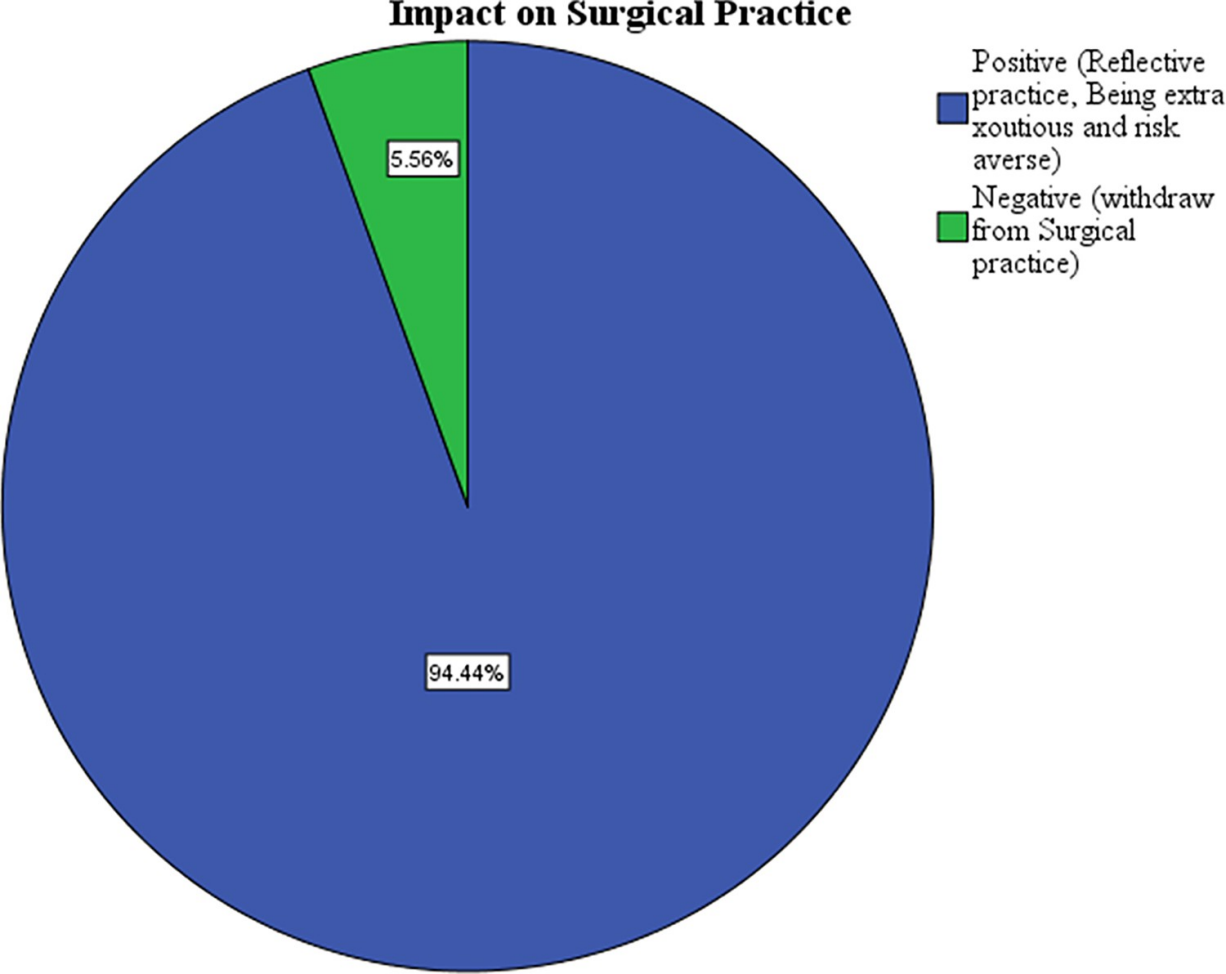
Impact on surgical practice of participants following the complications.

The occurrence of the complications had a positive impact on the surgical practice of the majority of the study participants (94.4%) (Fig 5).

### Predictors of a negative coping mechanism (blaming self or others, alcohol use, self-destruction, disassociation) following occurrence of a surgical complication

The independent predictors of having a negative coping mechanism were sex, worrying about reputation and guilt after occurrence of a complication. Females were 2.637 times more likely to have a negative coping mechanism compared to males (AOR = 2.637, CI 1.065–6.533, P = 0.036). Doctors who worried about reputation were 3.067 times more likely to use a negative coping mechanism compared to those who did not (AOR = 3.057, CI = 1.573–5.939, P = 0.001). Doctors who felt guilty following a complication were 4.417 times more likely to use a negative coping mechanism compared to those who did not (AOR = 4.417, CI = 2.253–8.659, P = <0.001) (Table 2). The results of the bivariate analysis are shown in S1 Table.

**Table 2:** Predictors of a negative coping mechanism (blaming self or others, alcohol use, self-destruction, disassociation) following occurrence of a complication.

Characteristic	Positive, N = 109 n(%)	Negative, N = 89 n(%)	Bivariate		Multivariate	
			P	COR(95% CI)	P	AOR(95% CI)
**Sex**						
Male **95(57.6)**		70(42.4)		Ref		
Female	14(42.4)	19(57.6)	0.113	1.842(0.865-3.924)	**0.036**	**2.637(1.065-6.533)**
**Worry about reputation**						
No	76(70.4)	32(29.6)		Ref		
Yes	33(36.7)	57(63.3)	<0.001	4.102(2.262-7.440)	**0.001**	**3.057(1.573-5.939)**
**Guilt**						
No	83(70.9)	34(29.1)		Ref		
Yes	26(32.1)	55(67.9)	<0.001	5.164(2.795-9.541)	**<0.001**	**4.417(2.253-8.659)**

## 4. Discussion

We set out to evaluate the psychosocial effects of postoperative surgical complications and the resilient coping mechanisms used by operating surgeons in their practice in selected teaching hospitals in Uganda and Eastern DRC because surgical complications are common, and their management is an integral part of surgical care, with impact on the operating surgeon, the "second victim," particularly in terms of psychological health [15].

Most of the participants in this study reported to be worried about the patient and their reputation and feeling guilt or disappointment as the commonest psychological impacts from postoperative complications among operating surgeons which are similar to findings from a scoping review by Subramanya in which depressive feelings, such as worry for the patient, guilt, anxiety, and disappointment, were more frequently expressed [1]. "Aggressive" and "depressive" emotions, which are frequently long-lasting and have an impact on other aspects of everyday life, are examples of the types of emotions described in literature [16].

Surgeons usually ask for help from friends, relatives, or colleagues once the patient’s issue has been handled [17]. In this study, more than half of participants used coping mechanisms which were considered positive such as discussing with colleagues, patients and relatives. Similarly, Biggs et al. observed that the majority of surgeons engaged with patients and families in open disclosure while discussing the technical details of cases with their peers [14]. Despite this, a high percentage of negative responses were reported by the operating surgeons in this study compared to other studies done elsewhere. Only a small percentage of people- (6% in the survey by Patel et al. and 10% in the study by [14] reported using harmful substances. According to the same study, 7% of surgeons had a propensity for dissociation, which could manifest as a reduction in social connections, avoidance, withholding, self-isolation, rumination, self-distraction and denial [14].

This study found that being female, worrying about reputation and guilt after occurrence of a complication were independent predictors of having a negative coping mechanism to postoperative surgical complications among participants, which were in relation to other studies.

A study review has demonstrated that differences in culture, educational opportunity, gender equity and women’s empowerment affect the experiences of both female surgical trainees and current female surgeons [18]. Female surgeons and young surgeons are more likely to personalize the situation, show more overt signs of being impacted, and be more forthcoming about this influence. These surgeons are reported to be overwhelmed due to imbalances between personal and professional lives [17]. In addition to that, a study done in Rwanda found out that biological basis for the gender disparity in surgery was reported by one of the female surgeons, stating that the difference was “testosterone and that men do not fear and female do fear [19]. Understanding these elements helps surgeons recognize the psychological reactions and vulnerabilities of the "second victim," which in turn helps them understand the coping strategy to use. Regardless of the severity of the issue, surgeons frequently believe that their technical proficiency and clinical judgment are to blame for complications [20,21]. Experience; attributing the problem to a lapse in judgement or focus, lack of knowledge or expertise, faults in the healthcare system are a few key factors that have been reported in several research in addition to being a woman, experiencing "burn-out", exhaustion, feeling demoralized or underappreciated, and seeing an imbalance between one’s personal and professional lives [21].

Surgery in such a situation may have sudden, unanticipated difficulties [22]. Given that self-criticism is a key predictor of depression in clinicians, this increased sense of personal responsibility may put surgeons at risk of experiencing extreme anguish after their involvement in major surgical problems [23]. It is known that 90% of complications may arise from 10% of patients and therefore, surgeons should investigate the “why” rather than the “what” after a surgical related complication [22]. Discussion with a senior colleague about the post-operative complications was found to be beneficial to avoid burnout among young surgeons [24]. Study found out that one possible solution for this barrier is to increase the mentorship and visibility of women in surgical specialties, which has been demonstrated in the US to positively influence young women to enter surgical specialties [25,26].

Because this study was retrospective in nature, it was limited by recall bias. Participants’ feelings or actions during problems or in the moments following a complication were not directly observed. Because of the unpredictable timing of difficulties and the unnecessary stress that such a study may put on the participants, a prospective design would be difficult.

## 5. Conclusion

Postoperative surgical complications continue to cause a huge psychological impact on the operating surgeon in developing countries such as Uganda and the Eastern DR Congo. Male surgeons experience it less frequently than do female surgeons. Despite complications following surgical operation, withdrawing from the career is not in the best interest of the health systems that already constrained. It is increasingly crucial now to understand that surgical complications might happen even in the best hands despite all precautions, and that in such a situation, the surgeon should get support and direction from colleagues. Operating surgeons might fully honor the art they have trained for if they recognized the value of excellent knowledge and training, good interpersonal relationships with patients, the team, and colleagues, good habits, and a healthy lifestyle.

## Supporting information

S1 Table Predictors of a negative coping mechanism (blaming self or others, alcohol, self-destruction, disassociation). (DOCX)

## References

[1] Siddaiah-subramanya M., To H., & Haigh C. (2021). The psychosocial impact of surgical complications on the operating surgeon: A scoping review. *Annals of Medicine and Surgery*, 67(June), 102530. 10.1016/j.amsu.2021.102530. 34276982 PMC8267492

[2] Regenbogen S. E., Veenstra C. M., Hawley S. T., Hendren S., Ward K. C., Kato I., et al. (2015). The effect of complications on the patient-surgeon relationship after colorectal cancer surgery. *Surgery*, 155(5), 841–850. 10.1016/j.surg.2013.12.011. PMC4254758 24787111

[3] Pinto A., Faiz O., Bicknell C., & Vincent C. (2013). Surgical complications and their implications for surgeons’ well-being. *British Journal of Surgery*, 100(13), 1748–1755. doi: 10.1002/bjs.9308 24227360

[4] Turner K., Johnson C., Thomas K., Bolderston H., & McDougal S. (2016). *The impact of complications and errors on surgeons*. 10.1308/rcsbull.2016.404.

[5] Dyrbye L., Satele D., Collicott P., Novotny P. J., & Sloan J. (2009). *Burnout and Career Satisfaction Among American Surgeons*. 250(3). 10.1097/SLA.0b013e3181ac4dfd. 19730177

[6] Scholar G. (2019). *Potential Consequences of Patient Complications for Surgeon Well-being A Systematic Review*. 1–7. 10.1001/jamasurg.2018.5640 30916741

[7] Mandowara B., Patel A. N., Amin A. A., Phatak A., & Desai S. (2020). Burden faced by caregivers of stroke patients who attend rural-based medical teaching hospital in western India. *Annals of Indian Academy of Neurology*. doi: 10.4103/aian.AIAN_406_18 32055120 PMC7001428

[8] Nundy S. (1999). Difficulties of surgery in the developing world: A personal view. *Lancet*, 353(SUPPL.1), 21–23. doi: 10.1016/s0140-6736(99)90225-8 10319928

[9] Albutt K., Punchak M., Kayima P., Namanya D. B., & Shrime M. G. (2019). Operative volume and surgical case distribution in Uganda’s public sector: A stratified randomized evaluation of nationwide surgical capacity. *BMC Health Services Research*, 19(1), 1–9. 10.1186/s12913-019-3920-9. 30728037 PMC6366061

[10] Ologunde R., Maruthappu M., Shanmugarajah K., & Shalhoub J. (2014). Surgical care in low and middle-income countries: Burden and barriers. *International Journal of Surgery*, 12(8), 858–863. doi: 10.1016/j.ijsu.2014.07.009 25019229

[11] Lavy C., Sauven K., & Mkandawire N. (2011). *State of Surgery in Tropical Africa*: *A Review*. 262–271. 10.1007/s00268-010-0885-6. 21153818

[12] Makumbi F., Galiwango E., Nordin P., Ibingira C., Forsberg B. C., & Wladis A. (2014). *Prevalence of treated and untreated groin hernia in eastern Uganda* 4 *¨*. 728–734. 10.1002/bjs.9457. 24652681

[13] Nthumba P., Solomkin J., Tarchini G., & Gibbs R. (2019). *Burden of surgical site infection following cesarean section in sub-Saharan Africa*: *a narrative review*. 309–318. 10.2147/IJWH.S182362 PMC6512794 31191039

[14] Biggs S., Waggett H. B., & Shabbir J. (2020). Impact of surgical complications on the operating surgeon. *Colorectal Disease*, 22(9), 1169–1174. doi: 10.1111/codi.15021 32065472

[15] Herring J.A.: Complications: Second Victim. J Pediatr Orthop., 2020; 40(Suppl 1): S22–S24. doi: 10.1097/BPO.0000000000001498 32502066

[16] Rothenberg W. A., Di Giunta L., Lansford J. E., Lunetti C., Fiasconaro I., Basili E., et al. (2019). Daily Associations between Emotions and Aggressive and Depressive Symptoms in Adolescence: The Mediating and Moderating Role of Emotion Dysregulation. *Journal of youth and adolescence*, 48(11), 2207–2221. doi: 10.1007/s10964-019-01071-6 31302795 PMC6993942

[17] Luu S., Patel P., St-Martin L. et al.: Waking up the next morning: surgeons’ emotional reactions to adverse events. Med Educ., 2012; 46(12): 1179–1188. doi: 10.1111/medu.12058 23171260

[18] Xepoleas M. D., Munabi N. C., Auslander A., Magee W. P., & Yao C. A. (2020). The experiences of female surgeons around the world: a scoping review. *Human resources for health*, 18, 1–28. 33115509 10.1186/s12960-020-00526-3 PMC7594298

[19] Yi S, Lin Y, Kansayisa G, Costas-Chavarri A. A qualitative study on perceptions of surgical careers in Rwanda: a gender-based approach. PLoS ONE. 2018;13(5):e0197290. doi: 10.1371/journal.pone.0197290 29746556 PMC5944995

[20] Patel A.M., Ingalls N.K., Mansour M.A. et al.: Collateral damage: the effect of patient complications on the surgeon’s psyche. Surgery, 2010; 148(4): 824–828; discussion 828–830. doi: 10.1016/j.surg.2010.07.024 20727563

[21] Seys D., Wu A.W., Van Gerven E. et al.: Health care professionals as second victims after adverse events: a systematic review. Eval Health Prof., 2013; 36(2): 135–162. doi: 10.1177/0163278712458918 22976126

[22] Bhattacharya K., & Bhattacharya N. (2022). Surgeon’s guilt after postoperative complication. *Polski Przeglad Chirurgiczny*, 94(4), 45–48. doi: 10.5604/01.3001.0015.6986 36047357

[23] Skevington SM, Langdon JE, Giddins G. ‘Skating on thin ice?’ Consultant surgeon’s contemporary experience of adverse surgical events. *Psychol Health Med* 2012; 17:1–16. doi: 10.1080/13548506.2011.592841 22191491

[24] Helo S., Moulton C.E.: Complications: acknowledging, managing, and coping with human error. Transl Androl Urol., 2017; 6(4): 773–782. doi: 10.21037/tau.2017.06.28 28904910 PMC5583051

[25] Neumayer L, Kaiser S, Anderson K, Barney L, Curet M, Jacobs D, et al. Perceptions of women medical students and their influence on career choice. Am J Surg. 2002;183(2):146–50. doi: 10.1016/s0002-9610(01)00863-7 11918878

[26] Xepoleas M. D., Munabi N. C., Auslander A., Magee W. P., & Yao C. A. (2020). The experiences of female surgeons around the world: a scoping review. *Human resources for health*, 18, 1–28. 33115509 10.1186/s12960-020-00526-3 PMC7594298

