# Supplementary material for: Psychosocial impact of surgical complications and the coping mechanisms among surgeons in Uganda and Eastern Democratic Republic of the Congo
Source: PLOS Glob Public Health. 2024 Apr 29;4(4):e0003180. doi: 10.1371/journal.pgph.0003180 (PMC11057973; doi:10.1371/journal.pgph.0003180)
Supplement: S1 Table — (DOCX) [file pgph.0003180.s001.docx]

# S1 Table. Predictors of a Negative Copying Mechanism (Blaming self or others, Alcohol, self-destruction, Disassociation)

| **Characteristic** | **Positive, N=109**  **n** | **Negative, N=89**  **n** | **Bivariate** | | | |
| --- | --- | --- | --- | --- | --- | --- |
|  |  |  | **P** | **COR** | **95% CI** | |
|  |  |  |  |  | **Lower** | **Upper** |
| **Age** | 34.7***** | 37.5***** | .024 | 1.042 | 1.005 | 1.079 |
| **Sex** |  |  |  |  |  |  |
| Male | 95 | 70 |  | Ref |  |  |
| Female | 14 | 19 | .113 | 1.842 | .865 | 3.924 |
| **Religion** |  |  |  |  |  |  |
| Christian | 87 | 85 |  | Ref |  |  |
| Non-Christian | 22 | 4 | .003 | .186 | .062 | .563 |
| **Grade** |  |  |  |  |  |  |
| Specialist | 23 | 22 |  | Ref |  |  |
| Resident | 71 | 44 | .221 | .648 | .323 | 1.298 |
| General Practitioner | 15 | 23 | .290 | 1.603 | .669 | 3.844 |
| **Position** |  |  |  |  |  |  |
| Hospital Director | 0 | 4 |  | N/A |  |  |
| HOD | 7 | 8 | .442 | 1.514 | .526 | 4.355 |
| Practitioner | 102 | 77 |  | Ref |  |  |
| **Sector** |  |  |  |  |  |  |
| Public | 56 | 56 |  | Ref |  |  |
| Private | 53 | 33 | .104 | .623 | .352 | 1.102 |
| **Years of experience** | 6.5 | 9.3 | .006 | 1.070 | 1.020 | 1.123 |
| **Only Surgeon in department** |  |  |  |  |  |  |
| No | 94 | 77 |  | Ref |  |  |
| Yes | 15 | 12 | .955 | .977 | .432 | 2.210 |
| **Full time** |  |  |  |  |  |  |
| Full Time | 91 | 81 |  | Ref |  |  |
| Part Time | 18 | 8 | .124 | .499 | .206 | 1.210 |
| **Specialty** |  |  |  |  |  |  |
| General surgery | 49 | 45 |  | Ref |  |  |
| Obstetrics and Gyn | 33 | 25 | .567 | .825 | .427 | 1.594 |
| Orthopedic Surgery | 22 | 10 | .105 | .495 | .212 | 1.158 |
| Neuro Surgery | 1 | 1 | .952 | 1.089 | .066 | 17.928 |
| Other | 4 | 8 | .228 | 2.178 | .614 | 7.729 |
| **Number of complications** |  |  |  |  |  |  |
| **<50** | 104 | 81 |  | Ref |  |  |
| **50-100** | 3 | 6 | .337 | 1.502 | .655 | 3.443 |
| **>100** | 2 | 2 | .337 | 1.502 | .655 | 3.443 |
| **Hemorrhage** |  |  |  |  |  |  |
| No | 22 | 7 |  | Ref |  |  |
| Yes | 87 | 82 | .018 | 2.962 | 1.201 | 7.304 |
| **SSI** |  |  |  |  |  |  |
| No | 26 | 13 |  | Ref |  |  |
| Yes | 83 | 76 | .107 | 1.831 | .878 | 3.819 |
| **Iatrogenic Injury** |  |  |  |  |  |  |
| No | 76 | 49 |  | Ref |  |  |
| Yes | 33 | 40 | .034 | 1.880 | 1.048 | 3.372 |
| **Death on table** |  |  |  |  |  |  |
| No | 79 | 65 |  | Ref |  |  |
| Yes | 30 | 24 | .930 | .972 | .518 | 1.824 |
| **High Spinal** |  |  |  |  |  |  |
| No | 89 | 69 |  | Ref |  |  |
| Yes | 20 | 20 | .473 | 1.290 | .644 | 2.584 |
| **Arrest on table** |  |  |  |  |  |  |
| No | 82 | 65 |  | Ref |  |  |
| Yes | 27 | 24 | .725 | .892 | .471 | 1.689 |
| **Others** |  |  |  |  |  |  |
| No | 104 | 86 |  | Ref |  |  |
| Yes | 5 | 3 | .667 | .726 | .169 | 3.123 |
| **Worry about reputation** |  |  |  |  |  |  |
| No | 76 | 32 |  | Ref |  |  |
| Yes | 33 | 57 | .000 | 4.102 | 2.262 | 7.440 |
| **Worry for patient** |  |  |  |  |  |  |
| No | 51 | 40 |  | Ref |  |  |
| Yes | 58 | 49 | .796 | 1.077 | .614 | 1.890 |
| **Anxiety** |  |  |  |  |  |  |
| No | 77 | 51 |  | Ref |  |  |
| Yes | 32 | 38 | .052 | 1.793 | .995 | 3.230 |
| **Guilt** |  |  |  |  |  |  |
| No | 83 | 34 |  | Ref |  |  |
| Yes | 26 | 55 | .000 | 5.164 | 2.795 | 9.541 |
| **Crisis of Confidence** |  |  |  |  |  |  |
| No | 87 | 52 |  | Ref |  |  |
| Yes | 22 | 37 | .001 | 2.814 | 1.499 | 5.281 |
| **Sadness** |  |  |  |  |  |  |
| No | 70 | 64 |  | Ref |  |  |
| Yes | 39 | 25 | .251 | .701 | .383 | 1.285 |
| **Disappointed** |  |  |  |  |  |  |
| No | 61 | 49 |  | Ref |  |  |
| Yes | 48 | 40 | .898 | 1.037 | .591 | 1.822 |
| **Anger** |  |  |  |  |  |  |
| No | 95 | 70 |  | Ref |  |  |
| Yes | 14 | 19 | .113 | 1.842 | .865 | 3.924 |

***= Group mean**
